# The evaluation of annuloplasty in bicuspid aortic valve repair using cardiac magnetic resonance

**DOI:** 10.1186/s12872-020-01831-4

**Published:** 2021-01-06

**Authors:** Marek J. Jasinski, Karol Miszalski-Jamka, Kinga Kosiorowska, Radoslaw Gocol, Izabella Wenzel-Jasinska, Grzegorz Bielicki, Mikolaj Berezowski, Marceli Lukaszewski, Andrzej Kansy, Marek A. Deja

**Affiliations:** 1 Department of Cardiac Surgery, University Hospital in Wroclaw, 50-556 Wrocław, Poland; 2 Division of Magnetic Resonance Imaging, Silesian Center for Heart Diseases, Zabrze, Poland; 3 Department of Cardiac Surgery, Medical University of Silesia, Katowice, Poland; 4 Silesian Medical College, Katowice, Poland; 5 Department of Cardiac Surgery, Children’s Memorial Paediatric Health Institute, Warsaw, Poland

**Keywords:** Aortic valve repair, Bicuspid aortic valve, Magnetic resonance imaging, External annuloplasty, Subcommissural annuloplasty

## Abstract

**Background:**

The incompetent bicuspid aortic valve (BAV) can be replaced or repaired using various surgical techniques. This study sought to assess the efficacy of external annuloplasty and postoperative reverse remodelling using cardiac magnetic resonance (CMR) and compare the results of external and subcommissural annuloplasty.

**Methods:**

Out of a total of 200 BAV repair performed between 2004 and 2018, 21 consecutive patients (median age 54 years) with regurgitation requiring valve repair with annuloplasty without concomitant aortic root surgery were prospectively referred for CMR and transthoracic echocardiography (TTE) one year after the operation. Two aortic annulus stabilization techniques were used: external, circumferential annuloplasty (EA), and subcommissural annuloplasty (SCA).

**Results:**

11 patients received EA and 10 patients were treated using SCA. There was no in-hospital mortality and all patients survived the follow-up period (median: 12.6 months (first quartile: 6.6; third quartile: 14.1). CMR showed strong correlation between postoperative aortic recurrent regurgitant fraction and left ventricular end-diastolic volume (r = 0.62; p = 0.003) as well as left ventricular ejection fraction (r = -0.53; p = 0.01). Patients treated with EA as compared with SCA had larger anatomic aortic valve area measured by CMR (3.5 (2.5; 4.0) vs. 2.5 cm^2^ (2.0; 3.4); p = 0.04). In both EA and SCA group, aortic valve area below 3.5 cm^2^ correlated with no regurgitation recurrency. EA (vs. SCA) was associated with lower peak transvalvular aortic gradients (10 (6; 17) vs. 21 mmHg (15; 27); p = 0.04).

**Conclusions:**

The repair of the bicuspid aortic valve provides significant postoperative reverse remodelling, provided no recurrent regurgitation and durable reduction annuloplasty can be achieved. EA is associated with lower transvalvular gradients and higher aortic valve area assessed by CMR, compared to SCA.

## Background

A bicuspid aortic valve (BAV) is the most common congenital cardiac abnormality affecting 1–2% of the general population. Even though significant regurgitation of the BAV is more common than that of the tri-leaflet aortic valve, it is also accompanied by aortopathy. Such a complex pathology of the bicuspid valve can be nowadays effectively repaired in selected patients [1]. To date, there are various techniques of an aortic valve repair described [2–5] and they are usually chosen based on a surgery-oriented classification of aortic regurgitation (AR) [6,7]. Of those, annular stabilization is one of the most important factors that may affect the mid-term and long-term results of the entire repair. However, there is no agreed consensus as to which of the annular stabilization techniques provides the best haemodynamic in patients with BAV.

Over the last few years, the role of cardiac magnetic resonance (CMR) in patients with aortic valve diseases is gradually increasing [8]. CMR is considered the current gold-standard non-invasive method for quantification of right- and left ventricular volumes, mass, global and regional systolic function. Moreover, CMR is able to assess aortic valve and aortic root morphology, mechanism of the dysfunction, and evaluate the degree of aortic regurgitation in a fully quantitative manner.

Therefore, the aim of this descriptive study was to analyse a prospective series of 21 patients in order to evaluate the predictors of postoperative reverse remodelling using CMR. Additionally, the study sought to compare in that respect different annuloplasty techniques: external annuloplasty in circumferential fashion and subcommissural annuloplasty.

## Methods

### Study population

Between 2004 and 2018, 200 consecutive patients (mean age: 43.2 ± 17.5 years; 148 males: 74%) underwent BAV repair at the same surgical setting (MJ), the results of which have already been published elsewhere [9,10]. Of these, 24 patients with BAV who developed aortic regurgitation requiring valve repair with annuloplasty without concomitant aortic root surgery were prospectively referred for CMR and transthoracic echocardiography (TTE) one year after the operation. Generally accepted contraindications to magnetic resonance imaging were used. Subsequently, 21 patients have had CMR performed. The size of the study and length of the follow-up were determined on the basis of a statistical power calculation of similar methods that were previously co-published by the main author [11]. This study was approved by the Local Ethics Committee. Informed consent was obtained from each subject.

### CMR acquisition protocol

All CMR examinations were performed on a 1.5 T clinical scanner (Signa HDxt, GE Healthcare, Milwaukee, WI). Images were obtained during suspended respiration at end-expiration. For cine imaging balanced steady-state free precession sequence was used. Patients underwent a left ventricular (LV) function study as previously described [9]. Cine images were acquired in three LV long-axis views, LV outflow tract view and a set of multiple contiguous short-axis slices from atrioventricular ring to the apex. Cine imaging of a BAV orifice was performed. The acquisition was repeated several times a few millimetres further and closer from valve orifice to ensure optimal direct planimetry of valve area. Then double oblique cine images were acquired as multiple cross-sections and long-axis views in order to visualize aortic root, sinotubular junction (STJ), and ascending aorta. After choosing a plane few millimetres above the tips of aortic cusps (usually at the level of STJ) 2D through-plane phase-contrast sequence was used for quantification of forward and backward flow. Then additional phase-contrast imaging was performed at the level of valve orifice and also few millimetres distal from tips of aortic cusps for maximal velocity assessment. Meticulous care was taken to orient imaging planes perpendicular to the blood flow. Encoding velocity was carefully adjusted to avoid aliasing. All flow sequences were acquired with the region of interest located at the isocenter of the magnet.

### CMR data analysis

Images were analysed with commercial software (Qmass MR, Medis Medical Imaging Systems, Leiden, the Netherlands) by an experienced, observer-blinded to the patients’ profile (KMJ). LV volumes, ejection fraction, and compacted mass were calculated as previously described [12]. Published sex-specific normal values were used to assess the normalization of LV parameters [13]. Three-chamber and LV outflow tract cine images were used for aortic annulus assessment. The average diameter of the annulus was calculated from minimal and maximal annulus diameters. Anatomical aortic valve area (AVA) planimetry was performed at the systolic frame (largest aortic valve opening) after careful confirmation of the correct imaging plane. Maximum cross-sectional measurements of the aortic root, STJ, and ascending aorta were performed. From phase-contrast images, forward and backward flow through the aortic valve was measured. Regurgitant volume and regurgitant fraction were calculated. In order to minimize phase offset errors background correction was applied with the region of interest in the stationary tissue (pectoralis muscle).

### Transthoracic echocardiography

All transthoracic echocardiograms were obtained one year after the surgery by an experienced, the same every time, observer-blinded to the patients’ profile. Standard echocardiographic parameters of the LV and aortic valve were assessed including the specific bicuspid anatomy, magnitude, and character of the aortic regurgitation, as well as transvalvular aortic gradients. Recommendations for non-invasive evaluation of valvular regurgitation developed by the American Society of Echocardiography in collaboration with the Society for Cardiovascular Magnetic Resonance has been used [14].

### Surgical management

All operations were performed through a median sternotomy, with the use of standard cardiopulmonary bypass. In each case, the myocardium was protected with blood cardioplegia. Techniques of aortic valve repair have been standardized and described previously by the author [10,15]. Briefly, the first step has been an evaluation of the effective height of leaflets [9,16], as well as the central leaflet coaptation. Secondly, the relative lengths of the leaflet free margins were assessed by suturing together and identifying prolapse [10,15,17–19]. The mechanism of AR was related to both, prolapse of conjoined fused leaflet and annulus enlargement. Prolapsing leaflet management consisted of plication with or without triangular excision followed by direct suturing. Annulus dilatation has been addressed either by external annuloplasty band or ring, or by subcommissural annuloplasty with regard to the height of the leaflets [17,20]. The postoperative assessment of successful repair was based on perioperative transoesophageal echocardiography (TOE), described elsewhere [10]. Briefly, postoperative valve evaluation included measurement of the aortic annulus, the presence of coaptation and assessment of effective coaptation height, as well as analysis of residual regurgitation, including quantification of regurgitation jet and its direction.

Twenty-four consecutive patients fulfilling preselection criteria operated on between 2013 and 2014 by a single surgeon (MJ), were prospectively allocated to one of two groups based on the aortic annulus stabilization technique. Three patients have been excluded due to CMR contraindications. The first group comprised patients who had an external annuloplasty (EA) in a circumferential fashion with the use of a Dacron strip made of aortic graft chosen for STJ remodelling and aorta replacement. The external annuloplasty consisted of the placement of a circular, transverse line of 6–8 interrupted pledgeted 2–0 braided sutures at the level of the aortic ring below leaflets nadirs, from inside to outside where were supported by a circular band from Dacron strip. The second group had subcommissural annuloplasty (SCA) performed with two braided 2–0 sutures enhanced with pledgets to narrow two subcommissural triangles. Additionally, all patients from the EA and SCA group underwent STJ remodelling and replacement of the ascending aorta.

The preselection criteria included: BAV type I (17 valves with left–right coronary cusp fusion and 4 valves with right-noncoronary cusp fusion, similarly distributed in both groups), moderate to severe aortic regurgitation, an enlarged aortic annulus (median: 26 mm (first quartile: 24; third quartile: 28)) with no aortic root dilatation (aortic root measurements below 40 mm), thus not requiring root replacement and valve reimplantation in accordance with the recommendations [21]. Both groups were comparable in terms of the baseline characteristics including patient age, dimensions of the left ventricle, and aortic annulus diameter. All patients presented with normal left ventricular contractility at baseline.

### Statistical analysis

Data are presented as counts (percentages) for categorical variables, and as medians with first and third quartiles of the distributions for continuous variables. Nonparametric tests were used due to the small sample size. For continuous variables, the Mann–Whitney test was performed for unpaired samples. Proportions comparison between groups were analysed with Fisher’s exact test. Correlations between continuous variables were evaluated with the use of Spearman’s rank correlation coefficient. All statistical tests were two-sided. A p-value was considered to indicate statistical significance at the nominal 0.05 level. Statistical analyses were performed using SPSS version 20.0 (SPSS Inc., Chicago, IL).

## Results

**Fig. 1:**
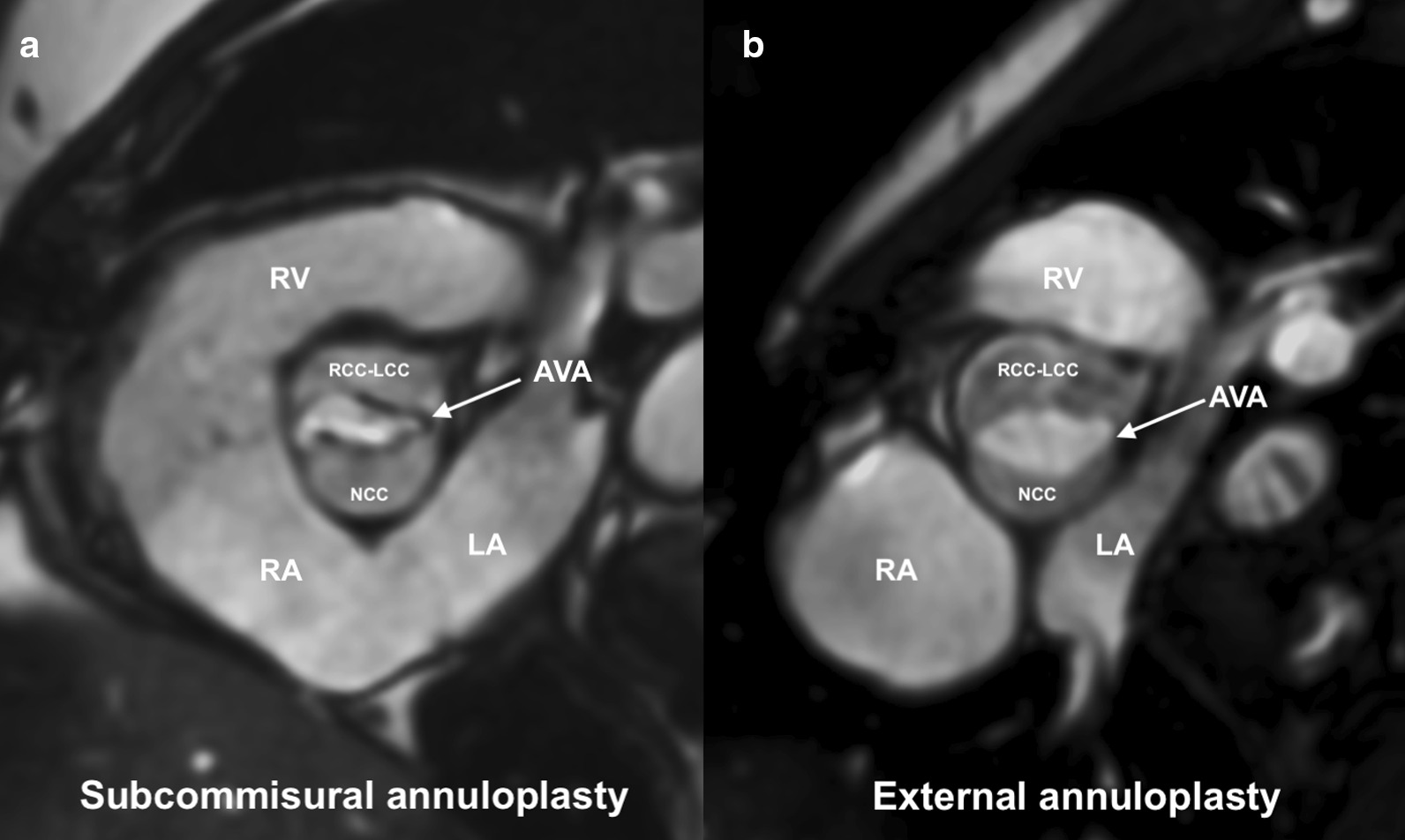
Cardiac magnetic resonance imaging of bicuspid aortic valve at end-systole in two patients treated with subcommissural annuloplasty (**a**) and external annuloplasty (**b**). After external annuloplasty (**b**) larger aortic valve area (AVA) was associated with lower transvalvular gradients as compared with subcommissural annuloplasty (**a**). AVA, aortic valve area; LA, left atrium; LCC, left coronary cusp; LV, left ventricle; NCC, noncoronary cusp; RA, right atrium; RCC, right coronary cusp; RV, right ventricle

Twenty-one Caucasian patients (median age 54 years (30; 65), 62% men) were included in the study. Of them, 11 patients received EA and 10 patients were treated using SCA. CMR examinations were acquired on average 12.6 months (6.6; 14.1) after the operation. In all patients, the quality of CMR images was sufficient for detailed analyses. Compared with patients who received SCA, EA-treated patients had wider aortic root diameter and larger AVA, which was also confirmed for the indexed values (Table 1). The latter group had also a lower transvalvular peak gradient measured by TTE (Fig. 1).

**Table 1:** Comparison of annuloplasty techniques using CMR and TTE (N = 21)

Characteristics	Overall (N = 21)	SCA (N = 10)	EA (N = 11)	P-value
*Demographic parameters*				
Age, years	53 (29; 66)	63 (31; 67)	50 (28; 63)	0.46
Gender male, n (%)	13 (62)	5 (50)	8 (73)	0.39
BSA, m^2^	2.0 (1.9; 2.2)	2.0 (2.0; 2.3)	1.9 (1.8; 2.2)	0.11
*CMR LV volume parameters*				
EDV, ml	186 (134; 237)	164 (131; 235)	193 (154; 242)	0.65
EDVI, ml/m^2^	89 (70; 114)	79 (66; 108)	95 (72; 121)	0.39
ESV, ml	68 (51; 118)	62 (47; 122)	76 (61; 116)	0.65
ESVI, ml/m^2^	35 (28; 55)	30 (24; 53)	36 (29; 57)	0.31
SV, ml	111 (84; 123)	93 (82; 124)	113 (91; 125)	0.56
SVI, ml/m^2^	53 (42; 64)	47 (40; 56)	55 (42; 65)	0.15
EF, %	59 (52; 62)	61 (49; 66)	58 (54; 61)	0.56
LV mass, g	115 (85; 151)	100 (85; 151)	116 (84; 158)	0.71
LV mass index, g/m^2^	53 (45; 72)	50 (44; 63)	63 (45; 83)	0.31
EDV normalization, n (%)	16 (76)	9 (90)	7 (64)	0.31
EF normalization (≥ 55%), n (%)	15 (71)	7 (70)	8 (73)	0.99
*CMR RV volume parameters*				
EDV, ml	170 (146; 196)	165 (140; 192)	173 (150; 203)	0.65
EDVI, ml/m^2^	83 (76; 96)	78 (71; 91)	91 (80; 98)	0.10
ESV, ml	72 (61; 84)	67 (60; 82)	73 (64; 91)	0.35
ESVI, ml/m^2^	35 (32; 43)	32 (29; 38)	38 (34; 48)	0.06
SV, ml	94 (79; 111)	92 (78; 111)	94 (84; 112)	0.81
SVI, ml/m^2^	47 (42; 53)	45 (39; 50)	48 (45; 55)	0.15
EF, %	56 (52; 61)	57 (55; 61)	55 (51; 62)	0.51
*CMR aortic valve/root parameters*				
Annulus, mm	22.0 (20.5; 26.8)	21.8 (19.8; 26.8)	22.5 (21.0; 27.0)	0.76
Annulus/BSA, mm/m^2^	10.9 (10.0; 13.7)	10.6 (10.0; 12.4)	12.7 (9.8; 14.1)	0.31
AVA, cm^2^	3.0 (2.1; 3.5)	2.5 (2.0; 3.4)	3.5 (2.5; 4.0)	**0.04**
AVA/BSA, cm^2^/m^2^	1.4 (1.1; 1.8)	1.1 (1.0; 1.5)	1.6 (1.3; 2.1)	**0.02**
Aortic root diameter, mm	38.4 (36.4; 41.6)	36.7 (35.8; 39.4)	39.8 (38.1; 43.8)	**0.02**
Aortic root diameter/BSA, mm/m^2^	19.7 (17.7; 21.7)	18.2 (15.9; 20.0)	21.5 (17.7; 24.2)	**0.03**
Aortic root height, mm	26.0 (23.5; 29.0)	25.5 (23.8; 28.5)	27.0 (23.0; 29.0)	0.61
STJ, mm	30.0 (28.0; 31.0)	30.0 (25.0; 30.0)	30.0 (29.0; 31.0)	0.20
Ascending aorta, mm	32.0 (30.5; 33.5)	32.0 (30.8; 33.3)	32.0 (30.0; 34.0)	0.81
Regurgitant fraction, %	7 (3; 12)	9 (3; 17)	6 (2; 12)	0.81
Regurgitant volume, ml	7 (3; 13)	7 (3; 20)	7 (2; 12)	0.81
Residual aortic regurgitation				0.51
None/mild, n (%)	17 (81)	8 (80)	9 (82)	
Moderate, n (%)	3 (14)	2 (20)	1 (9)	
Severe, n (%)	1 (5)	0 (0)	1 (9)	
*TTE parameters*				
Interventricular septal thickness at end-diastole, mm	12.0 (11.0; 13.3)	12.3 (11.6; 15.1)	11.5 (11.0; 13.0)	0.17
LV end-diastolic dimension, mm	53.0 (46.5; 59.3)	51.0 (44.0; 60.4)	54.0 (48.0; 58.0)	0.76
LV posterior wall thickness at end-diastole, mm	9.0 (8.0; 10.5)	9.5 (8.0; 11.3)	9.0 (7.0; 9.5)	0.25
Peak gradient, mmHg	16 (9; 24)	21 (15; 27)	10 (6; 17)	**0.04**
Residual aortic regurgitation				0.99
None/mild, n (%)	17 (81)	8 (80)	9 (82)	
Moderate, n (%)	4 (19)	2 (20)	2 (18)	
Severe, n (%)	0 (0)	0 (0)	0 (0)	

Values are presented as the number of patients (%) or median (first quartile; third quartile)

P-values below 0.05 were determined as statistically significant are shown in bold

AVA, aortic valve area; BSA, body surface area; CMR, cardiac magnetic resonance; EA, external annuloplasty; EDV, end-diastolic volume; EDVI, end-diastolic volume index; EF, ejection fraction; ESV, end-systolic volume; ESVI, end-systolic volume index, LV, left ventricle; RV, right ventricle; SCA, subcommissural annuloplasty; STJ, sinotubular junction; SV, stroke volume; SVI, stroke volume index; TTE, transthoracic echocardiography

**Fig. 2:**
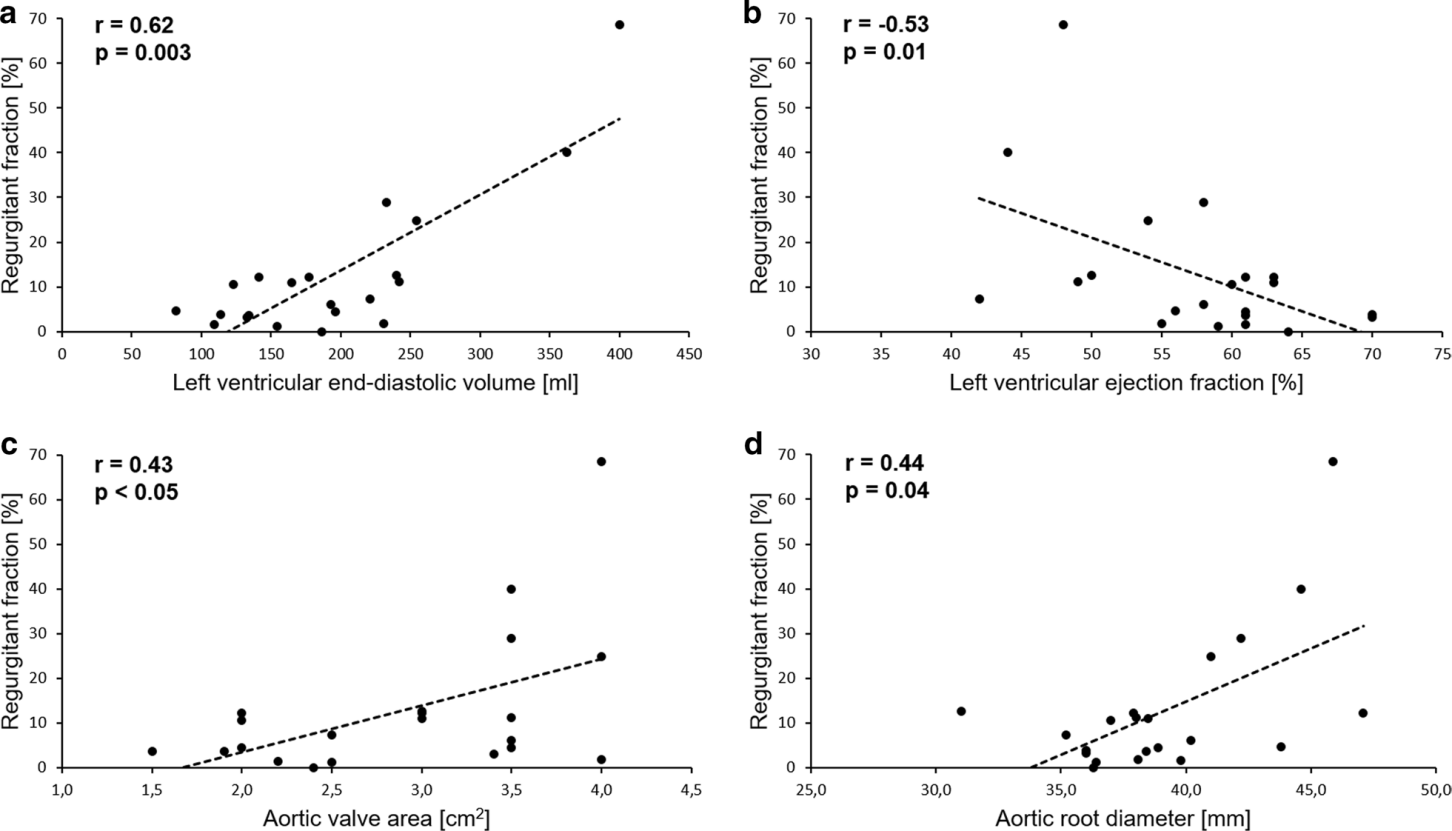
Correlations between severity of residual aortic regurgitation quantified by a regurgitant fraction and several parameters measured postoperatively using cardiac magnetic resonance: **a **left ventricular end-diastolic volume, **b **left ventricular ejection fraction, **c** anatomical aortic valve area, **d **aortic root diameter. The degree of each correlation is expressed with Spearman’s rank correlation coefficient (r) (N = 21)

There was a strong correlation between postoperative LV end-diastolic volume and severity of residual AR quantified by regurgitant fraction (r = 0.62; p = 0.003) and regurgitant volume (r = 0.66; p < 0.001). Similarly, the postoperative LV ejection fraction negatively correlated with the regurgitant fraction (r = − 0.53; p = 0.01) and the regurgitant volume (r = − 0.44; p = 0.04) (Fig. 2).

We also found a moderate correlation between the postoperative anatomical AVA and the severity of residual AR expressed by the regurgitant fraction (r = 0.43; p < 0.05) and the regurgitant volume (r = 0.42; p = 0.04). Similarly, the postoperative aortic root diameter is moderately correlated with the regurgitant fraction (r = 0.44; p = 0.04) and the regurgitant volume (r = 0.43; p = 0.04). However, no significant correlation was observed neither between the annulus diameter and the regurgitant fraction (r = 0.11; p = 0.63) nor between the annulus diameter and the regurgitant volume (r = 0.18; p = 0.45).

Of note, the postoperative transvalvular peak gradient measured by TTE significantly negatively correlated with anatomical AVA (r = −0.44; p = 0.04) and aortic root diameter (r = − 0.63; p = 0.002), but not with aortic annulus diameter (r = −0.20; p = 0.39).

The agreement between CMR and TTE for residual AR grading was excellent. Both methods yielded the same grade of AR severity in 20 of 21 patients (95%).

## Discussion

The presence of a bicuspid aortic valve is associated with a high incidence of valve dysfunction, proximal aortic dilatation, and a higher incidence of acute aortic events. Friedman et al. proved a very high incidence, up to 48—53%, of significant valve malfunction among the BAV population [22]. Moreover, Tzemos et al. observed cardiac events in 25% and aortic dilatation in 45% of 650 patients during a nine-year follow-up [23]. Similarly, in the study by Sarano et al. (Olmstead County asymptomatic BAV group) with a 15-year follow-up, 42% of patients experienced cardiac events, and 27% of the examined population required cardiac surgery [24]. Thanasoulis et al. found that predictors of aortic dilatation in 582 patients with BAV included moderate and severe AR, and right-left coronary cusp fusion [25]. The specific pattern of aorthopathy has been attributed to changes in aortic wall stress due to shear stress and different flow patterns [26]. Current recommendations advise the use of valve-sparing operations in patients with isolated AR [27]. However, the durability of the repair did not appear to be as good as with the tricuspid aortic valve. This may be related to a connective tissue disorder, which is often an accompanying feature of BAV [28]. We have published long-term data on aortic valve repair, confirming satisfactory long-term outcomes with 91.8% freedom from redo operations at 5 years [9,10].

Progressive annular dilatation caused by annuloaortic ectasia may affect the stability of the repair. Annular stabilization can be achieved by performing annuloplasty during the aortic valve repair operation. The annuloplasty techniques are currently under clinical investigation and it has already been confirmed that suture based SCA may fail due to late redilatation of the aortic root. Root stabilization with reimplantation provided better stability than SCA alone [29,30]. Other authors have also shown promising results of the internal or external band and ring [31,32]. Hence, our concept to analyse the CMR results of different annular stabilization strategies in BAV.

Our study suggests that external stabilization of the ventriculo-aortic junction provided better hemodynamic features, such as transvalvular gradients. Lower transvalvular aortic velocities and gradients after EA, compared to SCA, were associated with significantly larger AVA as measured using CMR. In our opinion, increased transaortic gradients and smaller AVA found in the SCA group may contribute to the future progression of aortic valve degeneration [33]. Using CMR with four-dimensional flow visualization, it has already been demonstrated that altered transvalvular flow patterns could normalize directly after BAV repair [34]. We believe that the choice of a durable annular stabilization technique is crucial to maintain normal transvalvular flow patterns.

Interestingly, the postoperative transvalvular peak gradient significantly negatively correlated with AVA and aortic root diameter, but not with aortic annulus diameter. Similarly, we found also a significant correlation between the postoperative severity of AR (expressed by a regurgitant fraction and regurgitant volume) with AVA and aortic root diameter, but not with annulus diameter. This suggests that a reduction in anatomical AVA derived from CMR may be a better marker of successful annuloplasty during BAV repair as compared with standard aortic annulus measurements.

Of note, anatomical AVA smaller than 3.5 cm^2^ correlated with no AR recurrency (Fig. 2). Similarly, aortic root diameter below 40 mm was a predictor of durable repair with no postoperative regurgitation. This supports the policy to routinely replace the aortic root during aortic valve repair when its diameter is above 40 mm. However, aortic measurements indexed to body surface area (BSA) should always be kept in mind as they are currently recommended as an additional parameter [35].

Our study also showed significant reverse remodeling after BAV repair. Normalization of LV ejection fraction and LV end-diastolic volume was achieved in the majority of the patient. However, no significant differences were observed between the two annular stabilization strategies. LV reverse remodeling is associated mainly with successful BAV repair considered as a lack of significant postoperative AR. The selection of the optimal time-point of operation is also crucial. In patients with moderate or severe AR, the CMR derived aortic regurgitant fraction > 33% and left ventricular end-diastolic volume > 246 ml were combined strongly associated with the development of symptoms or indications for surgery (with 85% sensitivity and 92% specificity) [36].

Currently, echocardiography remains the most established imaging modality for the assessment and follow-up of patients with valve diseases. However, over the last few years, the role of CMR in patients with valve diseases has been gradually increasing [8]. CMR is considered the current gold-standard method for the precise quantification of left and right ventricular volumes and systolic function. In comparison with echocardiography, a particular strength of CMR is the ability to assess the aortic regurgitant volume and regurgitant fraction in a fully quantitative manner with significantly higher reproducibility. This allows for adequate monitoring of disease progression over time. CMR may also be useful in assessing postoperative recurrence of AR. CMR assessment after bicuspid valve repair can be of great benefit due to the multifactorial and complex repair on one hand and the specificity and reproducibility of the CMR methodology compared to TTE on the other [37]. The 4D flow CMR may additionally add complex flow analysis and shear stress measurement leading to even more authentic and personalized assessment after aortic valve repair and valve-sparing aorta replacement [34,38].

We recognize that our study had limitations. Firstly, we performed a CMR one year after the operation. It has been demonstrated that after BAV repair transvalvular aortic gradients rapidly declined and then increased steadily over a long period of time (> 10 years) [33]. We cannot be absolutely sure that in the long-term EA would still demonstrate better haemodynamic compared to SCA. Secondly, the sample size is relatively small. However, due to the excellent quality of images and low inter-observer and inter-study variability, the use of CMR allows a significant reduction in sample size required to prove a research hypothesis in patients with AR. Specifically, CMR measurement of LV volume parameters (i.e. EDV, ESV, and EF) [39] and AR severity (i.e. regurgitant volume) is significantly less variable than with echocardiography [40]. Compared to two-dimensional echocardiography, CMR was shown to reduce the required sample size by several times, even by 81–97% [39], making the size of our group comparable to similar studies and of adequate statistical power [11].

## Conclusion

In conclusion, CMR appears to be a very promising tool for assessing BAV repair and subsequent reverse remodelling of the left ventricle. EA in circumferential fashion is associated with lower transvalvular gradient and higher AVA compared to SCA. A reduction in anatomical AVA derived from CMR may be a better marker of successful annuloplasty during BAV repair as compared with standard aortic annulus measurements.

We believe that the results of repair of the BAV may be further improved with more aggressive root stabilization, especially at the level of the ventriculo-aortic junction.

## Data Availability

The datasets used and/or analysed during the current study are available from the corresponding author on reasonable request.

## Abbreviations

AR: Aortic regurgitation
AVA: Aortic valve area
BAV: Bicuspid aortic valve
BSA: Body surface area
CMR: Cardiac magnetic resonance
EA: External annuloplasty
LV: Left ventricle
SCA: Subcommissural annuloplasty
STJ: Sino-tubular junction
TTE: Transthoracic echocardiography
TOE: Transoesophageal echocardiography
