# Correction to: The evaluation of annuloplasty in bicuspid aortic valve repair using cardiac magnetic resonance

**DOI:** 10.1186/s12872-021-01912-y

**Published:** 2021-02-15

**Authors:** Marek J. Jasinski, Karol Miszalski-Jamka, Kinga Kosiorowska, Radoslaw Gocol, Izabella Wenzel-Jasinska, Grzegorz Bielicki, Mikolaj Berezowski, Marceli Lukaszewski, Andrzej Kansy, Marek A. Deja

**Affiliations:** 1 Department of Cardiac Surgery, Wroclaw Medical University, Wrocław, Poland; 2 Division of Magnetic Resonance Imaging, Silesian Center for Heart Diseases, Zabrze, Poland; 3 Department of Cardiac Surgery, Medical University of Silesia, Katowice, Poland; 4 Silesian Medical College, Katowice, Poland; 5 Department of Cardiac Surgery, Children’s Memorial Paediatric Health Institute, Warsaw, Poland

## Correction to: Jasinski et al. BMC Cardiovasc Disord https://doi.org/10.1186/s12872-020-01831-4

Following publication of the original article [1], the authors identify an error in affiliation 1:

The incorrect affiliation is: Department of Cardiac Surgery, University Hospital in Wroclaw, 50–556, Wrocław, Poland.

The correct affiliation is: Department of Cardiac Surgery, Wroclaw Medical University, Wroclaw, Poland.
